# Exploring the relationship between neurologists and older persons with multiple sclerosis through the lens of social support theory

**DOI:** 10.1177/20552173241281458

**Published:** 2024-10-03

**Authors:** Mina Stanikić, Felix Gille, Jonas Schlomberg, Paola Daniore, Susanne Kägi, Andrew Chan, Christian P Kamm, Chiara Zecca, Pasquale Calabrese, Patrick Roth, Claudia Baum, Irene Rapold, Milo A Puhan, Viktor von Wyl

**Affiliations:** Epidemiology, Biostatistics and Prevention Institute, University of Zurich (UZH), Zurich, Switzerland; Institute for Implementation Science in Health Care, University of Zurich (UZH), Zurich, Switzerland; Institute for Implementation Science in Health Care, University of Zurich (UZH), Zurich, Switzerland; Digital Society Initiative, University of Zurich (UZH), Zurich, Switzerland; Institute for Implementation Science in Health Care, University of Zurich (UZH), Zurich, Switzerland; Institute for Implementation Science in Health Care, University of Zurich (UZH), Zurich, Switzerland; Digital Society Initiative, University of Zurich (UZH), Zurich, Switzerland; Swiss Multiple Sclerosis Society, Zurich, Switzerland; Department of Neurology, Inselspital, Bern University Hospital, University of Bern, Bern, Switzerland; Department of Neurology, Inselspital, Bern University Hospital, University of Bern, Bern, Switzerland; Neurocentre, Lucerne Cantonal Hospital, Lucerne, Switzerland; Department of Neurology, Neurocenter of Southern Switzerland, Ospedale Regionale di Lugano, EOC, Lugano, Switzerland; Faculty of Biomedical Sciences, 27216 Università della Svizzera Italiana, Lugano, Switzerland; Neuropsychology and Behavioral Neurology Unit, Division of Cognitive and Molecular Neuroscience, 27209 University of Basel, Basel, Switzerland; Department of Neurology and Brain Tumor Center, University Hospital and University of Zurich, Zurich, Switzerland; Rehabilitation Clinic Zihlschlacht, Zihlschlacht, Switzerland; Citizen Scientist; Epidemiology, Biostatistics and Prevention Institute, University of Zurich (UZH), Zurich, Switzerland; Epidemiology, Biostatistics and Prevention Institute, University of Zurich (UZH), Zurich, Switzerland; Institute for Implementation Science in Health Care, University of Zurich (UZH), Zurich, Switzerland

**Keywords:** Multiple sclerosis, social support, doctor–patient relationship, patient perspective, aging

## Abstract

**Background:**

Although healthcare practitioners (HCPs) are a valuable source of social support, research on support provided by neurologists to older persons with multiple sclerosis (pwMS) remains limited.

**Objectives:**

To explore expectations of pwMS aged 55 years or older regarding MS care and to identify support types, met and unmet needs within their relationship with neurologists.

**Methods:**

Utilizing a mixed-methods approach, we analyzed survey data from Swiss Multiple Sclerosis Registry participants. Quantitative data included Likert scales gauging the importance of various aspects of MS care for pwMS both in and out of neurological care. Qualitative data were derived from three open-ended questions, focusing on neurologist-provided support for pwMS in neurological care. Data underwent descriptive and deductive thematic analysis, using Cutrona and Suhr framework for coding social support.

**Results:**

Among the 286 participants (median age 61.0 years, interquartile range (IQR) 57.0–66.0; median disease duration 23.5 years, IQR 15.0–31.0), 84.6% (*N* = 244) were under neurological care. Quantitative findings highlighted the significance of HCP expertise and consultation time. Qualitative analysis identified all social support domains in the neurologist–pwMS relationship, with informational support being most prevalent, followed by emotional support. Neurologists’ expertise, availability, comprehensive advising, listening, and validation emerged as key themes. Unmet needs were relatively infrequent and concerned insufficient information on complementary medicine, empathy, and understanding of symptoms like fatigue.

**Conclusions:**

Older pwMS see neurologists as adequate providers of comprehensive support and particularly value neurologists’ sufficient availability, informational and emotional support. Areas for improvement include attention to complementary medicine and empathy.

## Introduction

Healthcare professionals (HCPs) have the potential to contribute to individuals’ well-being by providing emotional, informational and tangible support.^1^ These support types fall under the umbrella term of social support, which encompasses perceived support, i.e. the perception of the availability of support and the actual supportive behaviors in interpersonal relationships.^2,3^

Social support has been recognized as a critical factor contributing to the improved health-related quality of life of older persons with multiple sclerosis (pwMS), with potential to offset deficiencies in other aspects associated with healthy ageing.^4^ Research consistently underscores the significance of both emotional and informational support from HCPs for many pwMS, including those in geriatric and palliative care, who may have unique concerns and needs.^5–8^ Older pwMS attach great importance to the emotional support provided by HCPs, demonstrating a willingness to actively seek out HCPs who can offer emotional support alongside medical assistance and informational support.^9^ Furthermore, older pwMS value two-way communication and collaborative relationship with HCPs, perceiving ineffective listening by HCPs as unhelpful.^10^

Among all HCPs in MS care, neurologists retain a pivotal role, guiding treatment decisions and serving as a primary source of information for pwMS.^11^ While support provision is identified as one of the core roles of neurologists by pwMS,^12^ understanding of the support within the neurologist–patient relationship in MS care is limited and predominantly derived from studies that investigated social support from all sources or all HCPs involved in MS care, rather than the support specifically provided by neurologists.

To address the lack of research on neurologist-provided support for older pwMS, we conducted an exploratory mixed methods study within the Swiss Multiple Sclerosis Registry (SMSR). First, we aimed to quantitatively explore expectations of older pwMS regarding care and services provided by MS clinics and HCPs to assess general interest in support provision. Next, we aimed to qualitatively explore perceived social support provided by neurologists to pwMS in neurological care by identifying types of support as well as met and unmet support needs within the neurologist–pwMS relationship, using the social support coding framework by Cutrona and Suhr.^13^

## Methods

A mixed-methods approach was employed to descriptively analyze data derived from multiple-choice and open-ended survey responses.

### Study population and data source

We used data from participants enrolled in the SMSR, an ongoing observational study in adults with MS residing or receiving treatment in Switzerland. The SMSR employs the citizen science approach to determine focus topics and develop questionnaires for the collection of self-reported data. Participation in the registry is voluntary and requires the completion of a consent form along with confirmation of MS diagnosis. The SMSR was approved by the Ethics Committee of the Canton of Zurich (PB-2016–00894; BASEC-NR 2019-01027). Additional information about the SMSR can be found elsewhere.^14,15^

Both quantitative and qualitative data utilized in this study primarily originate from the SMSR follow-up questionnaire released in November 2021. In addition to the questions on the disease status, medication usage and other MS and health-related questions typically included in the SMSR follow-up questionnaires, this questionnaire also included questions about participants’ expectations and the significance they assigned to services provided by MS practices and HCPs, along with open-ended questions on perceived support provided by neurologists. These questions were informed based on insights from a July 2021 focus group workshop involving five SMSR participants, aimed at exploring the importance of various dimensions of MS care to pwMS. The invitation to complete the follow-up questionnaire was sent to all active participants. However, this study only included participants aged 55 years or older at the time of questionnaire completion who responded to at least one question of interest for this study. Quantitative analysis included all participants meeting the specified criteria, whereas qualitative analysis involved only those currently undergoing neurological treatment. Additionally, we utilized sociodemographic data from the baseline SMSR questionnaire.

### Quantitative data collection and analysis

Quantitative data concerned participants’ sociodemographic, MS-related, and health-related characteristics. Information on age, sex (female or male), Swiss citizenship (yes or no), highest education level (university degree, higher professional education, mandatory, high school, and apprenticeship), marital status (married and in a registered partnership or not), and gait disability (categorical, mild, moderate, or severe) as measured by Self-reported Disability Status Scale,^16^ was collected in the SMSR baseline questionnaire. The duration of MS in years was calculated based on the year of diagnosis collected at baseline, up until the completion date of the follow-up questionnaire used in this study. The SMSR follow-up questionnaire included self-reported MS type, previously shown to have satisfactory validity and reliability^17^ (relapsing-remitting MS (RRMS), primary progressive MS, secondary progressive MS (SPMS), clinically isolated syndrome, and transition between RRMS and SPMS), and a question regarding current treatment by a neurologist (yes or no). These data were used to describe the study sample and were analyzed using descriptive statistics, reporting frequencies, medians, or means.

The follow-up questionnaire included two Likert scales to evaluate participants’ expectations and the importance they attributed to services and care provided by MS practices and HCPs. The statements in the questions were developed using input from SMSR participants provided during the focus group workshop. Each statement reflects aspects of care that were identified and collectively agreed upon by the focus group participants as key elements of care provision in clinics, or as essential qualities of HCPs. The Likert scales comprised five response options: “not important at all,” “less important,” “no opinion,” “important,” and “very important.” Participants were instructed to select the response option that best aligned with their perception of the construct's importance. Additionally, participants were asked to evaluate the support they receive from their neurologists on a scale ranging from 1 to 10, using a slide-bar. These questions served as an introduction to the open-ended questions about neurologist-provided support and were analyzed descriptively, whereby the different care aspects were ranked by their perceived importance.

All questions utilized in the quantitative analysis are provided in the Supplemental material. All quantitative analyses were conducted in R and R studio.^18^

### Qualitative data collection

Qualitative data were collected through three open-ended questions specifically addressing the support provided by a neurologist. To best reflect current standards in neurological care and avoid recall bias, these questions were only accessible to participants who indicated ongoing neurological treatment. Participants were first prompted to describe the type of relationship they aspired to have with their neurologist. They were then asked to identify the most valuable aspects of support provided by their neurologist and provide reasons for their choices. Finally, participants were given the opportunity to express whether there was any additional support they would have liked to receive from their neurologists. Questions are provided in the Supplemental material.

Data were collected in Switzerland’s three official languages: German, Italian, and French. To ensure confidentiality, anonymized data from the three open-ended questions were automatically translated for analysis into English using the DeepL Pro desktop version for iOS. Prior to translation, the data underwent a thorough review to ensure the absence of any identifying information that could compromise anonymity. Translated data were manually inspected for quality, and occasional spot checks were conducted by a native speaker or a coauthor with native-level proficiency in the respective language.

### Qualitative data analysis

Applying deductive thematic analysis, we explored and categorized types of support reported by the participants, as well as their fulfilled and unmet support needs, using the framework developed by Cutrona and Suhr.^13^ The framework comprises five main categories (informational, emotional, tangible, esteem, and network support) and 23 subcategories and has previously been employed in pwMS.^19^ To ensure the applicability of the framework to the local context of the MS community, we adapted the definitions of the subcategories of support in a collaborative process with an SMSR participant and citizen scientist who was over 55 years of age (IR).

Data from all three questions on support were independently coded by two coders (MS and JS). The results were then compared to establish a consistent and reliable coding procedure, and any discrepancies in codes were resolved through discussion between the coders. The first author then revisited the data through an iterative process to ensure adherence to the established coding consensus. Any data that did not fit into the predefined categories of the adopted coding framework were analyzed to identify new themes.

Finally, we provided a quantitative overview of the qualitatively analyzed data by displaying the numbers and frequencies of codes for the different types of support. Codes falling within the same support category were counted only once per participant.

Data analysis was conducted using the MAXQDA software for qualitative data analysis.^20^

## Results

### Sample description and quantitative analysis

A total of 2238 invitations were sent to SMSR participants, of which 786 were aged 55 or older at the time of invitation. Of these invitations, 1016 participants responded to the survey, of whom 302 were 55 years of age or older. This represents a response rate of 45.4% for the entire cohort and 38.4% for those aged 55 years and over. The median age of nonresponders was 62.0 years with interquartile range (IQR) of 58.0 to 68.0 years, and 66.2% of them were women.

**Figure 1.**
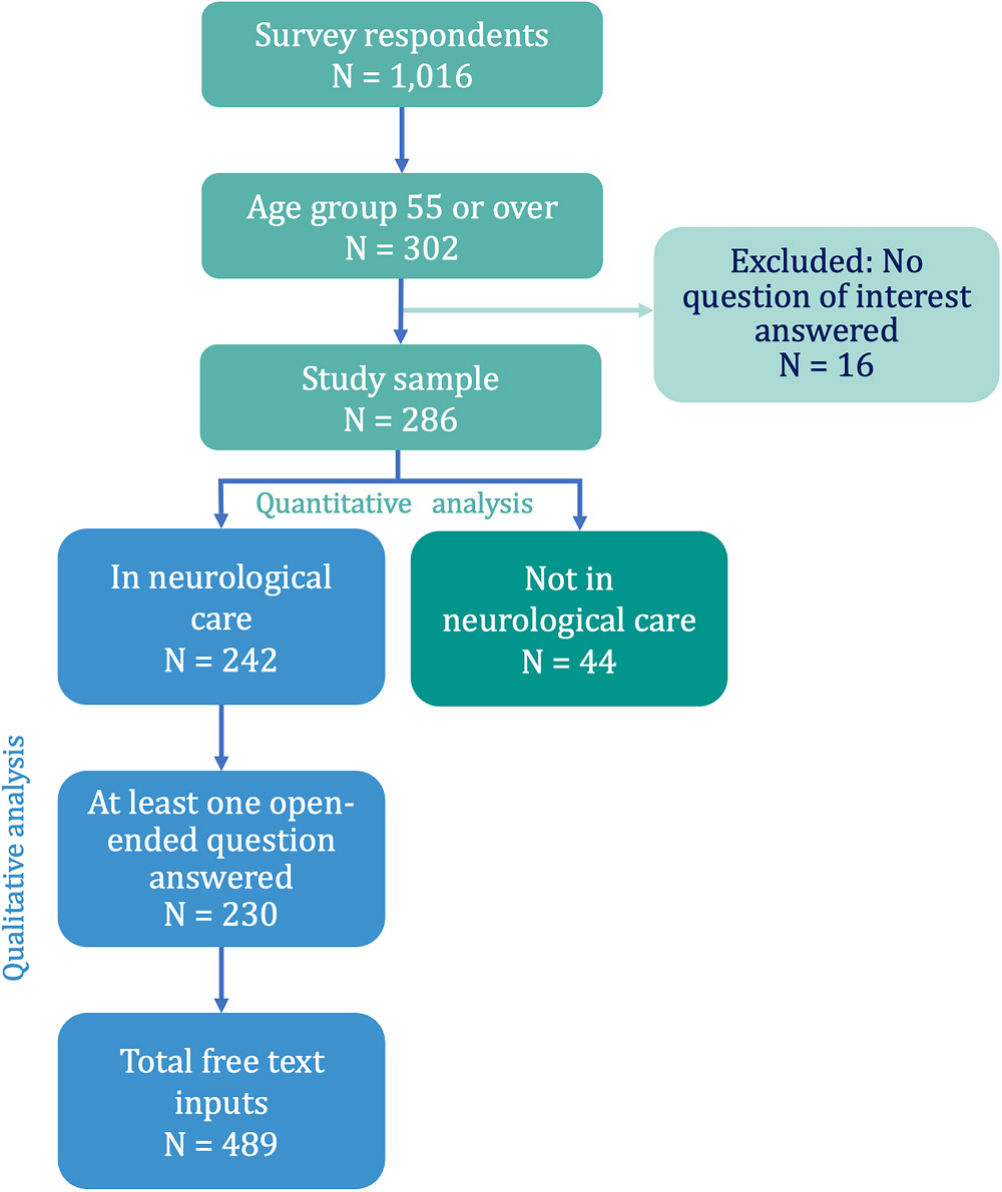
Study design flowchart.

The quantitative analysis included data from 286 participants aged 55 or older at the time of questionnaire completion, among whom 44 participants (15.4%) were not receiving neurological care (Figure 1). The median age was 61.0 years, with an IQR of 57.0 to 66.0 years. Two-thirds of the participants were female (*N* = 191, 66.8%), and the majority had RRMS (*N* = 138, 48.3%). Participants who were not receiving neurological care were more frequently female (*N* = 34, 77.3% compared to *N* = 157, 64.9%), older (median age of 64.5, IQR 61.0 to 70.0 years compared to 60.0, IQR 34.0 to 56.0 years), and had been living with MS for a longer duration compared to those who were receiving neurological care (median of 23.5, IQR 18.3 to 38.3 years compared to median of 21.0, IQR 15.0 to 30.0 years) (Table 1). Participants who were in neurological care rated the neurologist-provided support with a median score of 8.0 (IQR 7.0 to 9.0), out of a maximum score of 10.0.

**Table 1.** Socio-demographic, health and MS-related characteristics of the study sample.

Characteristic	In neurological care (*N* = 242)	Not in neurological care (*N* = 44)	Overall (*N* = 286)
Age			
Median (interquartile range)	60.0 [57.0, 65.0]	64.5 [61.0, 70.0]	61.0 [57.0, 66.0]
Sex, *N* (%)			
Female	157 (64.9%)	34 (77.3%)	191 (66.8%)
Male	85 (35.1%)	10 (22.7%)	95 (33.2%)
Years since diagnosis			
Mean (standard deviation)	22.4 (10.7)	27.7 (11.9)	23.3 (11.1)
Median (interquartile range)	21.0 [15.0, 30.0]	23.5 [18.3, 38.3]	22.0 [15.0, 31.0]
Missing	20 (8.3%)	2 (4.5%)	22 (7.7%)
MS^a^ type, *N* (%)			
CIS^b^	1 (0.4%)	1 (2.3%)	2 (0.7%)
PPMS^c^	31 (12.8%)	7 (15.9%)	38 (13.3%)
RRMS^d^	120 (49.6%)	18 (40.9%)	138 (48.3%)
SPMS^e^	90 (37.2%)	17 (38.6%)	107 (37.4%)
Transition	0 (0%)	1 (2.3%)	1 (0.3%)
Gait disability, *N* (%)			
Mild	27 (61.4%)	127 (52.5%)	154 (53.8%)
Moderate	10 (22.7%)	85 (35.1%)	95 (33.2%)
Severe	6 (13.6%)	27 (11.2%)	33 (11.5%)
Missing	1 (2.3%)	3 (1.2%)	4 (1.4%)
Swiss citizenship, *N* (%)	221 (91.3%)	41 (93.2%)	262 (91.6%)
Missing	2 (0.8%)	0 (0%)	2 (0.7%)
Marital status, *N* (%)			
Divorced or separated	39 (16.1%)	11 (25.0%)	50 (17.5%)
Married or in registered partnership	153 (63.2%)	22 (50.0%)	175 (61.2%)
Unmarried	36 (14.9%)	7 (15.9%)	43 (15.0%)
Widowed	6 (2.5%)	3 (6.8%)	9 (3.1%)
Missing	8 (3.3%)	1 (2.3%)	9 (3.1%)
Highest education level, *N* (%)			
Higher professional education	34 (14.0%)	7 (15.9%)	41 (14.3%)
Mandatory, high school or apprenticeship	125 (51.7%)	26 (59.1%)	151 (52.8%)
University or applied university	74 (30.6%)	10 (22.7%)	84 (29.4%)
Missing	9 (3.7%)	1 (2.3%)	10 (3.5%)
Seeing neurologist, *N* (%)			
In private practice	106 (43.8%)	NA	106 (43.8%)
In hospital	112 (46.3%)	NA	112 (46.3%)
Both	11 (4.5%)	NA	11 (4.5%)
Missing	13 (5.6%)	NA	13 (5.6%)

^a^ multiple sclerosis, ^b^ clinically isolated syndrome, ^c^ relapsing-remitting multiple sclerosis, ^d^ primary progressive multiple sclerosis, ^e^ secondary progressive multiple sclerosis.

**Figure 2.**
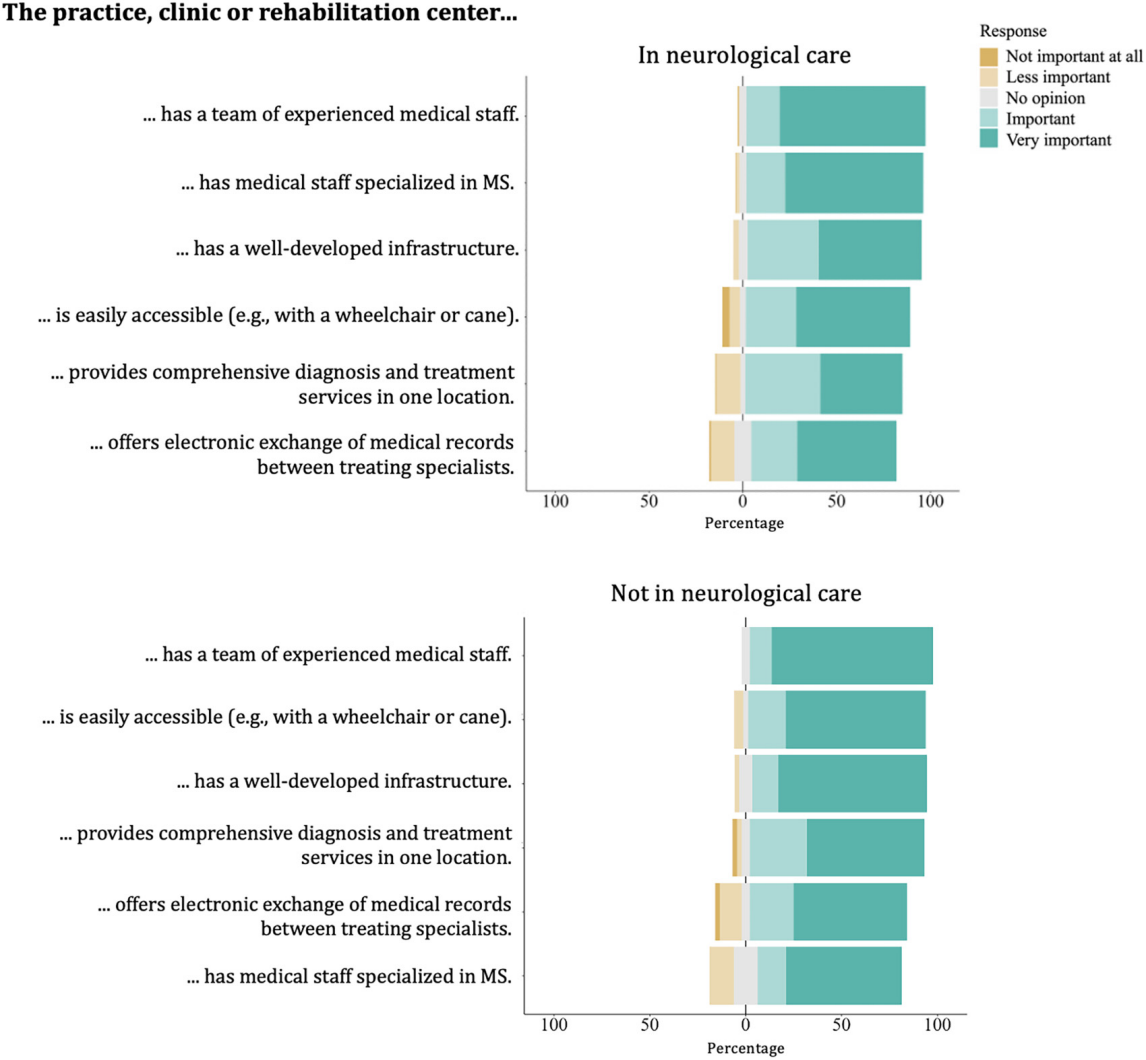
Participants’ ratings of the importance of services and infrastructure in practices, clinics, and rehabilitation centers.

**Figure 3.**
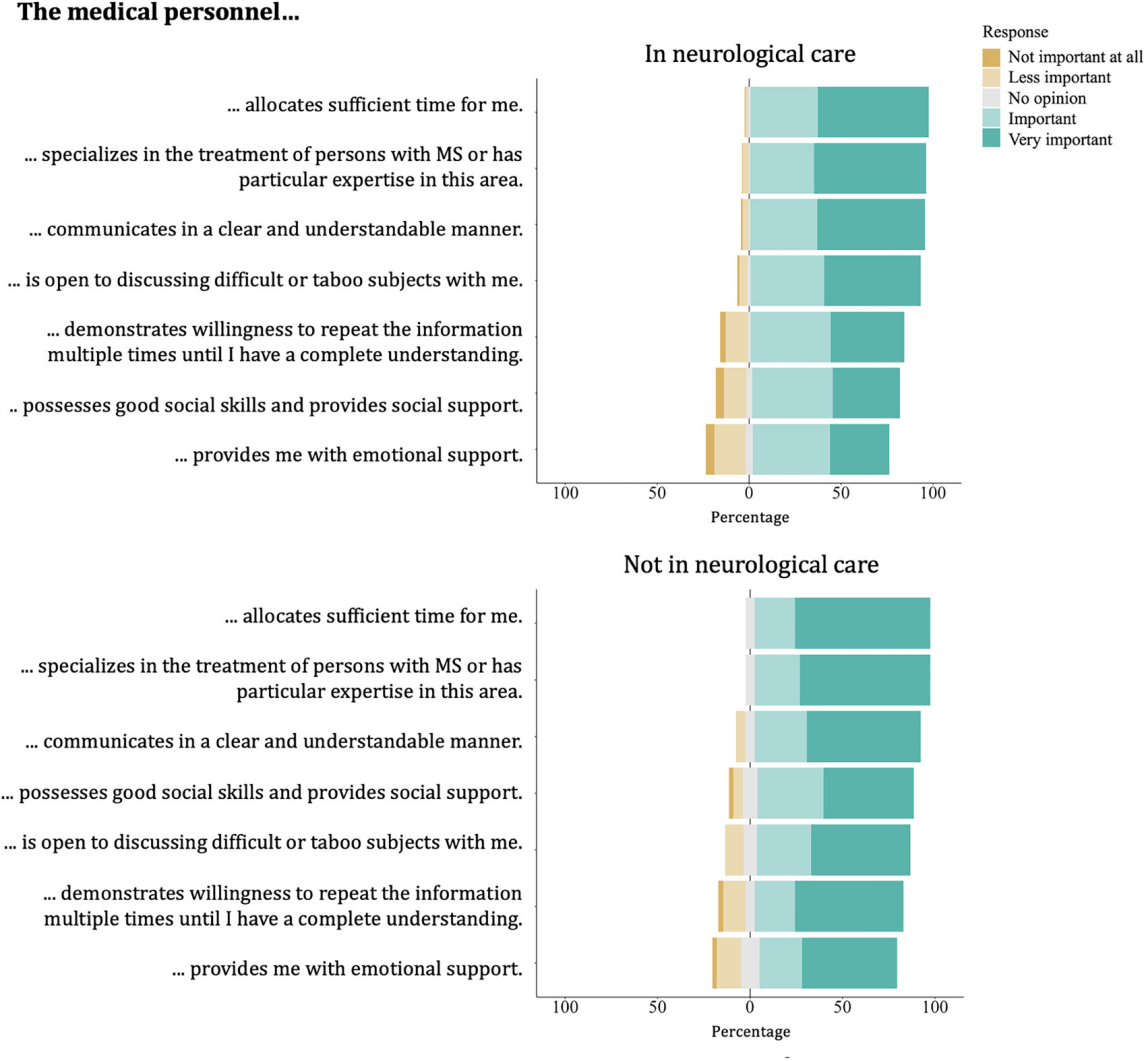
Participants’ ratings of the importance of the qualities and expertise of healthcare professionals in providing MS care.

Figure 2 illustrates the distribution of importance ratings given by participants to services and infrastructure in practices, clinics, and rehabilitation centers. Participants receiving neurological care prioritized experience and expertise in MS of the practice staff over practical aspects such as infrastructure. For participants not receiving neurological care, easy access to the practice or clinic was also very important. Figure 3 shows the distribution of importance participants attached to qualities and expertise of HCPs in MS care. Participants rated the allocation of sufficient time by HCPs and their MS expertise as most important, while the provision of emotional support by the HCPs was perceived as comparatively less important. Supplemental material provides visual analysis of Likert scale questions by MS type subgroup, as well as mean scores for each statement by subgroups of sex, age, MS duration, MS type, and gait disability.

MS: multiple sclerosis.

### Identified dimensions of support and met needs in the neurologist–pwMS relationship

**Figure 4.**
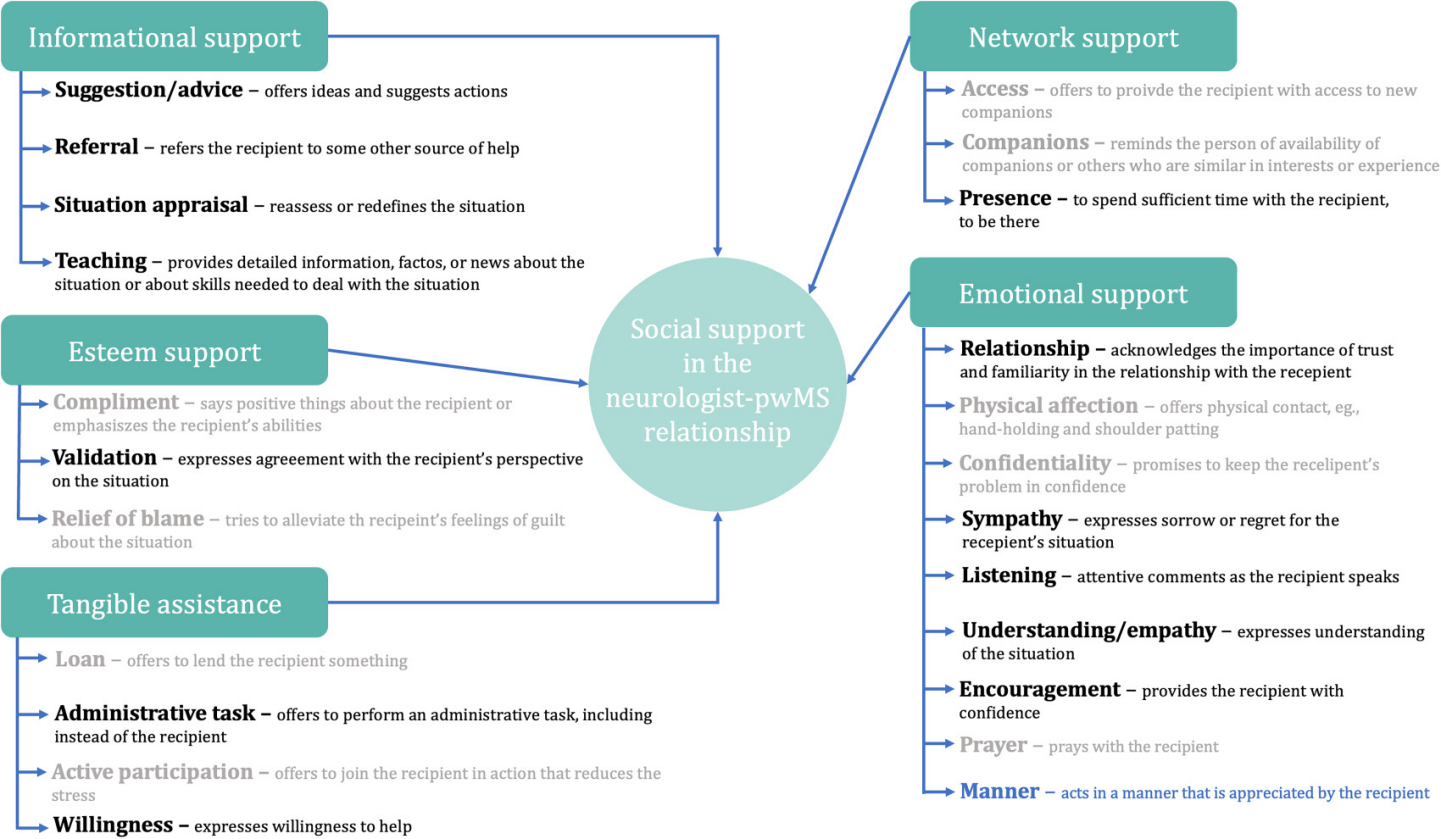
Adapted social support coding framework by Cutrona and Suhr, incorporating context-modified descriptions of the purpose of supportive behaviors. The dimensions of social support identified in the relationship between neurologists and older persons with multiple sclerosis (pwMS) are represented in black. In **light gray**, we indicated dimensions of support not identified in our study. Additionally, based on thematic analysis of the collected data, we introduced a new dimension of support, represented in **blue**.

We identified all five support dimensions following Cutrona and Suhr's framework^13^ in 489 free-text inputs from 230 participants who answered at least one open-ended question (Figure 1). However, some framework proposed subcategories were not supported by the data. Figure 4 illustrates both identified and unsupported subcategories along with contextual adaptations and explanations.

Among the five support dimensions outlined by the framework, informational support was most frequently mentioned, with 187 mentions from 123 participants, followed by emotional support, identified 105 times in inputs from 78 participants. Esteem support was the least prevalent category, mentioned in 26 instances by 21 participants. Supplemental material offers an overview of the frequency of codes by sex and MS type.

Table 2 presents participants’ perceived support and met needs across support categories and subcategories, narratively summarizing key themes alongside example quotes and brief keyword summaries. Identified themes were found to be interconnected, with similar themes recurring across multiple subcategories (e.g., perception of expertise, clear communication, and shared decision-making in teaching, advice, and suggestions). Subcategories were also interdependent, with factors such as continuous presence facilitating long-term relationships and trust, and sufficient consultation time enabling patient inquiries to be addressed effectively.

*"Through the long-standing doctor–patient relationship, we know that we can trust each other."*

**Table 2.** Overview of identified types of perceived support and met support needs in the relationship between older pwMS and neurologists.

**Support category**	**Subcategory**	**Narrative summary**	**Quote**	**Bullet point summary**
**Informational**	Suggestion/ advice	Participants appreciated neurologists who offered expert advice extending beyond MS management to cover job retention and financial matters, reflecting their desire for comprehensive care. Particularly valued was neurologists’ proactive problem-solving approach, presenting multiple options and tailored suggestions without imposition, meeting individual needs. Importance of dialogue and open communication between the neurologist and the participants was notably evident in the context of shared decision-making regarding therapies.	*“[My neurologist] is also a contact person in financial questions, procurement of aids, etc.”* *“[My neurologist] presents various possibilities and collaboratively determines the course of action during discussions.”*	Expertise-based advice Comprehensive care Proactive problem-solving Tailored, nuanced advice
	Teaching	Teaching relied on neurologists’ demonstrated expertise and professionalism, evident in thorough consultation preparation, clear communication, and staying updated on MS research, especially regarding new treatments. Participants valued neurologists’ openness about lacking knowledge and their commitment to obtaining information, seeing it as professionalism and effective teaching. They highly valued the opportunity to ask questions, express concerns, and receive honest, prompt, detailed, and understandable answers. Similar to suggestion and advice giving, open communication and dialogue were perceived as important for successful teaching.	*“[My neurologist] admits when she doesn't know something and seeks out the desired information to help me further.”*	Expertise and professionalism Clear communication Valued participant engagement
	Situation appraisal	Participants positively viewed the neurologists’ proactive approach to assessing their current condition, especially through thorough questioning and examinations. Participants were of the perception that successful situation appraisal led to neurologists adjusting treatment, advice, and suggestions accordingly, or referring participants to other sources of help when needed.	*“[I appreciate that my neurologists adapt] treatments when she hears or reads something ‘between the lines’.”*	Proactive assessment Disease management adjustment
	Referral	Participants generally felt that referral was only necessary for “major” problems, regardless of the type of help to which the participant was referred.	*“[My neurologist] listens to my problems and if [those] were bigger problems, she would refer me to a psychologist.”*	Appropriate referrals
**Esteem**	Validation	Validation consisted of participants’ viewpoints and perspectives being treated with respect, with neurologists attentively addressing and taking their symptoms and concerns seriously, engaging in conversations on an equitable “eye-level.”	*“Since I have myself worked in nursing for about 35 years, we can really talk to each other at eye level. Above all, she takes me seriously.”*	Respectful validation Attentiveness toward complaints Equitable conversations
**Tangible**	Administrative task/willingness	Performing an administrative task or expressing willingness to perform it was reflected in the exchange of information between neurologists, other specialists, and primary care physicians. Additionally, neurologists offered to liaise with health insurance companies to ensure or facilitate reimbursement for treatments, diagnostic tests, or walking aids.	*“For me, it is very important that my family doctor exchanges with neurologists. I can rely on them to play together.”*	Exchange with other HCPs Liaison with insurers
**Network**	Presence	Participants appreciated the neurologists’ ample availability, which went beyond sufficient time during scheduled appointments to include direct communication channels such as phone or email, bypassing intermediaries like practice employees or nurses and enabling a quick response. Moreover, their consistent long-term availability, along with their demonstrated patience, was seen as a valued advantage.	*“My neurologist takes a lot of time during my visits; I never have the feeling of being ‘dispatched’.”* *“I can call [my neurologist] at any time, even if the complaints are not directly related to MS. I can get an answer directly from him in the shortest time.”*	Multiple communication channels Quick response Long-term availability
**Emotional**	Relationship	Familiarity and reliability were key components within the “relationship” subcategory. Familiarity, cultivated over time, formed the foundation of the neurologist-pwMS relationship. This extended connection allowed neurologists to gain a deep understanding of individual medical concerns and fostered patient comfort and trust.	*“[My neurologist] has known my ‘problems’ for years, always shows understanding and offers assistance.”*	Familiarity Reliability Long-term relationship
	Listening	Listening was the most frequently mentioned aspect of emotional support, with active listening, including responses and recognition, being particularly valued.	*“What I really appreciate about my neurologist is that she listens to me and, as possible, also states things clearly and her response time to questions and problems is short.”*	Attentive and active listening
	Understanding/ empathy	Participants highly valued when their neurologists showed understanding and expressed empathetic behavior.	*"My neurologist is highly empathetic; she can also provide praise or express regret when something is not going well anymore."*	Showing understanding and empathy
	Sympathy	The distinction between sympathy and empathy was blurred, with participants more frequently underscoring the importance of empathy, while only one participant explicitly praised their neurologist for being sympathetic.	*“[My neurologist] has a sympathetic ear.”*	Sympathetic behavior
	Encouragement	Neurologists offered encouragement through affirmative actions and by refraining from displaying a negative attitude toward the disease or its prognosis.	*“[My neurologist] listens, motivates me and always suggests that I try therapies and exercises. Compared to other neurologists, she's not fatalistic.”*	Affirmative actions Avoidance of negative attitude
	Manner	We recognized “manner” as a distinct subcategory of emotional support, acting as a mediator that enhances the recipient's favorable perception of all other subcategories. Positive elements of the neurologists’ demeanor encompass kindness, warmth, and attentiveness. The overall perception of exhibiting “humane” behavior was highly regarded. Additionally, there were commendations for neurologists who incorporated balanced humor, optimism, and cheerfulness into their communication.	*“I am satisfied with my neurologist's sober, scientific style, but he can also show humor.”*	Warmth Kindness “Humane” approach Humor, cheerfulness

pwMS: persons with multiple sclerosis.

### Unmet support needs in the neurologist-pwMS relationship

Unmet needs within the support domains were relatively uncommon compared to met needs. Present in the data of 36 participants, the most commonly observed unmet need was for informational support, followed by emotional and network support, each mentioned by 11 participants. Similar to met needs, there was an interdependency between support domains, notably prominent with insufficient time allocation or the absence of long-term availability of the same neurologist resulting in lacking emotional support.

*“As the doctors always change, the emotional support also stays out.”*

Table 3 presents an overview of key themes concerning unmet needs, including a call for improved educational and advisory services, communication tailored for lay comprehension, and the integration of teaching on complementary medicine. Additionally, there was an emphasized need for heightened empathy and increased availability of neurologists.

**Table 3.** Overview of identified types of unmet support needs in the relationship between older pwMS and neurologists.

**Support category**	**Subcategory**	**Narrative summary**	**Quote**	**Bullet point summary**
**Informational**	Suggestion/ advice	Participants called for more comprehensive care and advice that go beyond pharmaceutical interventions targeting disease activity to cover mental health concerns and preventive actions.	*“More help in organizing my day-to-day life, giving me addresses and places where I can find help concerning the facilities that disabled people can have (parking card, address of the Swiss Multiple Sclerosis Society, car tax…). We have very little support, we fight our way through the professional jungle, insurance and so on, all on our own, when we're already very busy with MS.”*	More comprehensive and holistic care Mental health and prevention advice
	Teaching	Unclear explanations or communication that surpassed lay comprehension, along with information dissemination lacking explicit actionable recommendations, were viewed negatively. Notably, there was a pronounced demand for insights into complementary medicine, such as acupuncture, and its potential benefits, which participants hope to receive from neurologists.	*“[I wish] more input in terms of complementary medicine treatment options [from my neurologist].”* *“I would like to [get] clearer answers with appropriate explanations, and … that fears and concerns are addressed.”*	Unclear communication Nonactionable guidance Teaching about complementary medicine
**Esteem**	Validation	Participants expressed a desire for greater validation of their experiences with symptoms, particularly fatigue, and neurologists’ recognition of their perspective on which symptoms are attributable to MS.	*“Sometimes, I'd like [my neurologist] to put herself more in my shoes, especially when it comes to fatigue. I have the impression that at this point, she doesn't understand my exhaustion at work, which is rather physical.”*	Validation of patients’ perception
**Tangible**	Administrative task/willingness	Unmet needs for tangible assistance primarily involved participants’ desire for improved assistance in communicating with insurance companies and obtaining prescriptions and reports.	*“[It] often takes a bit long for [a] report to be available. Report is often delivered only after request.”*	Assistance with insurers Assistance with prescriptions
**Network**	Presence	Limited access to their neurologist between visits and the perception of the neurologist's distraction during appointments was seen as an unmet need. Participants also expressed concerns centered on the concept of changing doctors, with the inability to see the same doctor for follow-up visits being viewed unfavorably.	*“[I wish my neurologist] had more time. You can't just talk to an employee on the phone. The employee tells the neurologist. Neurologist gives the answer, and the employee then tells you. It's too complicated and as a patient you feel a bit left alone at that moment.”* *“It would be nice not to have a new doctor every year, where you have to tell everything again for yet another time…”*	Regular consultations Focused presence Long-term availability
**Emotional**	Understanding/ empathy	Participants expressed a need for greater empathy, both at the time of the initial diagnosis and during subsequent follow-up visits, as well as for neurologists to show understanding.	*“Higher empathy; I have noticed that when I am not well (e.g., new relapse) [my neurologist] does not handle it well.”*	Greater and continuous empathy Showing understanding
	Encouragement	Participants desired neurologists to provide a supportive response when patients communicated their needs and concerns.	*“More supportive words when I describe something as it is at the moment and what I hope for.”*	Supportive language

pwMS: persons with multiple sclerosis.

### Absence of need for emotional support

Finally, 21 participants explicitly stated that they did not need emotional support. Their perspective is rooted in their preference for the neurologist's primary role to be focused on medical guidance. Mild disease course, satisfaction with support from friends and family, and a preference for keeping emotional matters private in interactions with HCPs contributed to the participants’ lack of need for neurologist-provided emotional support. Supplemental material shows characteristics of these participants.

*“Since my MS has been calm since the first relapses and I have only minor complaints, I do not need any emotional support-I feel very well taken care of by my wife and family.”*

## Discussion

In this mixed-methods study, we analyzed survey data from 286 participants aged 55 years or older in the SMSR. Participants rated the importance of MS care aspects and provided free-text insights into the support provided by their neurologists. Quantitative analysis showed that participants prioritized HCP expertise and sufficient time for consultations, with less emphasis on HCP-provided emotional support and practical services. Qualitative analysis focused exclusively on the relationship with the neurologists and largely mirrored the quantitative results by highlighting the importance of sufficient consultation time and informational support. Emotional support from neurologists, characterized by empathy and understanding, was also a highly valued aspect of support in the qualitative analysis. Finally, instances of unmet needs were relatively uncommon across support categories, with informational and emotional support representing primary areas for improvement.

Neurologists are regarded as the preferred source of information for pwMS,^11^ who in turn recognize information provision as a core role of neurologists.^12^ Similarly, among various information sources, older pwMS predominantly seek informational support from HCPs.^9^ Our findings confirm this trend, with participants rating HCP information provision as paramount and emphasizing the significance of informational support from neurologists, particularly in educational aspects. Informational support was the most favorable when it was expertise-based, using understandable language, and providing actionable guidance and comprehensive care that includes information relevant to daily life with MS. This aligns with earlier research indicating that older pwMS expressed concern over the lack of specificity in neurologists’ instructions^6^ and valued HCPs who not only facilitated access to information and services but also offered guidance on day-to-day issues.^8^ Furthermore, neurologists who actively sought patient input were commended for considering patients’ perspectives and engaging in shared decision-making, addressing not only treatment-related decisions but also other aspects such as employment.

A recognized gap in HCP-provided informational support for pwMS includes information on disease course, treatment options, available services, diet, exercise, and complementary medicine.^21,22^ In our study, the most frequently cited unmet information need was for complementary medicine, alongside mental health and prevention. Increased interest in complementary medicine, less emphasized in prior research, may stem from the limited range of disease-modifying therapeutic options available to older pwMS, prompting exploration of alternative treatments. Effective information provision, along with motivation, especially by neurologists, is crucial as it can positively influence behavioral changes in pwMS, improving their physical, psychological, and social well-being.^23–25^ However, constraints like time, training gaps, and insurance issues have been identified as barriers for promoting exercise among neurologists,^26^ and may similarly affect other aspects of comprehensive informational support, such as offering guidance on complementary medicine. Therefore, involving other HCPs in MS care, such as MS nurses, occupational therapists, or physiotherapists, could address the unmet informational needs of pwMS, ensuring comprehensive care delivery.

Thus far, there has been limited understanding of more subtle support aspects, such as emotional and esteem support. Although HCP-provided emotional support ranked lower in importance in quantitative analysis, in qualitative analysis neurologist-provided emotional support emerged as highly valued. Our participants valued encouragement, understanding, and empathy from their neurologists, with a minority expressing a desire for increased empathy, aligning with previously identified unmet needs.^6^ Neurologists and physicians caring for pwMS perceive physical functions, such as mobility and ambulation, as the most important bodily function,^27^ leading them to prioritize treatment of physical symptoms.^28^ As a result, less attention may be given to providing emotional support. However, according to our findings, neurologist–pwMS relationships focusing solely on medical treatment can be satisfactory, especially for those with milder disease courses or strong social support from other sources.

Regardless of disease course or emotional support needs, the availability of neurologists, continuous care, and remote communication were pivotal in fostering effective relationships between older pwMS and neurologists. Approachable HCPs and readily available services were previously found to be appreciated by older pwMS.^4^ Additionally, HCPs who supported older pwMS in acquiring knowledge and confidence contributed to strengthening patients’ self-management over time.^10^ While the notion of neurologists providing hope, as previously observed in individuals with disabling MS,^12^ was not evident in our study, encouragement, motivation, validation, and respectful treatment further fortified the doctor–patient relationship and mutual trust. Previously, persons severely affected by MS expressed a desire for increased respect from physicians.^21^ Although this did not surface as an unmet need, our study highlighted a desire for supportive language and validation of participants’ perceptions of symptoms, particularly regarding fatigue. Considering that fatigue has been recognized as one of pwMS’ major concerns,^28^ dedicating greater attention to its management may lead to improvements in patient-reported outcomes.

Finally, several subcategories of support proposed by the framework, such as relief of blame, compliments, or physical affection, were not observed in our data either as met or unmet needs. This suggests that the support provided by neurologists is not exhaustive, nor is it expected to be. Neurologist support constitutes only a component of overall support and should not replace support from other relationships, including both other healthcare professionals involved in MS care, as well as friends and family, which are recognized as crucial sources of support for pwMS.^29–31^

### Strengths and limitations

To our knowledge, this study is the first to exclusively explore neurologist support for older pwMS, enabling detailed investigation of the specific needs of older pwMS. By integrating quantitative and qualitative methods and analyzing data from over 200 participants, we ensured data saturation and the comprehensiveness of our findings. Nonetheless, there are limitations to this study. The use of exploratory rather than validated questionnaires and scales to assess expectations about MS care and to measure support may have provided a less reliable assessment of these aspects. Additionally, the COVID-19 pandemic and the sequence of the questionnaire, with support-related questions placed at the end, may have contributed to selection bias, affecting response rates and completion. The pandemic may also have influenced participants’ perceptions of support from their neurologists, especially since some consultations may have been conducted online; however, no evidence of such influence was supported by the data. Lastly, our findings are shaped by Switzerland's healthcare landscape, including virtually universal insurance coverage and healthcare access. Certain findings, such as unmet needs related to changing neurologists, may be context-specific.

## Conclusions

Older pwMS see neurologists as adequate providers of informational, esteem, network, emotional, and tangible support. Older pwMS value neurologists who provide comprehensive care, bringing expertise, guidance, and teaching, coupled with attentive listening and understanding. Continuous care, regular consultations, and remote communication are key in forming mutual trust and a reassuring doctor-patient relationship. Unmet needs, primarily insufficient information on complementary medicine, empathy, and understanding of symptoms like fatigue, are relatively uncommon within support categories. Our findings can aid neurologists in identifying and addressing support gaps to meet their patients’ needs.

## Supplemental Material

sj-docx-1-mso-10.1177_20552173241281458 - Supplemental material for Exploring the relationship between neurologists and older persons with multiple sclerosis through the lens of social support theory Supplemental material, sj-docx-1-mso-10.1177_20552173241281458 for Exploring the relationship between neurologists and older persons with multiple sclerosis through the lens of social support theory by Mina Stanikić, Felix Gille, Jonas Schlomberg, Paola Daniore, Susanne Kägi, Andrew Chan, Christian P Kamm, Chiara Zecca, Pasquale Calabrese, Patrick Roth, Claudia Baum, Irene Rapold, Milo A Puhan and Viktor von Wyl in Multiple Sclerosis Journal – Experimental, Translational and Clinical
