# Supplementary material for: Exploring the relationship between neurologists and older persons with multiple sclerosis through the lens of social support theory
Source: Mult Scler J Exp Transl Clin. 2024 Oct 3;10(4):20552173241281458. doi: 10.1177/20552173241281458 (PMC11475095; doi:10.1177/20552173241281458)
Supplement: sj-docx-1-mso-10.1177_20552173241281458 - Supplemental material for Exploring the relationship between neurologists and older persons with multiple sclerosis through the lens of social support theory [file sj-docx-1-mso-10.1177_20552173241281458.docx]

*Supplementary material*

*Third COVID-19 SMSR questionnaire*

Since the outbreak of the *COVID-19* pandemic, the Swiss Multiple Sclerosis Registry (SMSR) has conducted three shorter surveys related to COVID-19, in addition to regular follow-up surveys. The present study used data from the third such survey, which was available to SMSR participants in German, French and Italian. Only the first page and the questions used in this study are included in this **Supplementary material** and have been translated into English for the purpose of this publication only.

To circumvent potential confusion around the term “social support”, often confused with “social services” in the Swiss context, we intentionally refrained from employing the term "social support" in the open-ended questions. Anticipating that participants would naturally encompass informational support within their responses, the initial question emphasized the aspect of emotional support and included an explanation with examples such as "through listening, encouragement, or showing empathy." The subsequent two questions utilized the term "support" without further specification.

**Third supplementary survey on Coronavirus**

**
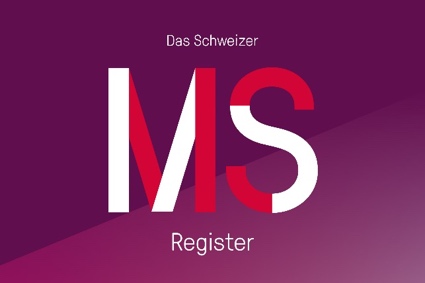
**

**Swiss MS Register**

**2021**

**Part D: Support and satisfaction in the context of medical MS treatment.**

*Below are some questions about satisfaction with MS medical care and what you consider to be good support from the treating neurologist.*

| **D1.** | **How important do you find the following practical aspects of MS care in the practice, clinic or rehabilitation center?**  *Please select all that apply.* |
| --- | --- |

| **The practice, clinic or rehabilitation center...** | |  | | **Not important at all** | |  | | **Little important** | |  | | **Important** | |  | | **Very important** | |  | | **No opinion** | |  |
| --- | --- | --- | --- | --- | --- | --- | --- | --- | --- | --- | --- | --- | --- | --- | --- | --- | --- | --- | --- | --- | --- | --- |
|  | ... is easy to reach (e.g. with a wheelchair or cane). | |  | | O | |  | | O | |  | | O | |  | | O | |  | | O | |
|  | ... has a good infrastructure. | |  | | O | |  | | O | |  | | O | |  | | O | |  | | O | |
|  | ... has experienced medical professionals. | |  | | O | |  | | O | |  | | O | |  | | O | |  | | O | |
|  | ... has MS-specialized medical professionals. | |  | | O | |  | | O | |  | | O | |  | | O | |  | | O | |
|  | ... enables all diagnoses and treatments to be carried out in one place. | |  | | O | |  | | O | |  | | O | |  | | O | |  | | O | |
|  | ... offers the electronic exchange of medical history between treating professionals. | |  | | O | |  | | O | |  | | O | |  | | O | |  | | O | |

| **D2.** | **How important do you think the following aspects are in relation to the medical staff treating you? (doctors, nurses, physiotherapists, etc.)?**  *Please select all that apply.* |
| --- | --- |

| **The medical staff...** | |  | | **Not important at all** | |  | | **Little important** | |  | | **Important** | |  | | **Very important** | |  | | **No opinion** | |  |
| --- | --- | --- | --- | --- | --- | --- | --- | --- | --- | --- | --- | --- | --- | --- | --- | --- | --- | --- | --- | --- | --- | --- |
|  | ... is specialized or particularly experienced in the treatment of people with MS. | |  | | O | |  | | O | |  | | O | |  | | O | |  | | O | |
|  | ... has enough time for me. | |  | | O | |  | | O | |  | | O | |  | | O | |  | | O | |
|  | ... has an understandable language / expression. | |  | | O | |  | | O | |  | | O | |  | | O | |  | | O | |
|  | ... Is ready to repeat the information several times until I fully understand it. | |  | | O | |  | | O | |  | | O | |  | | O | |  | | O | |
|  | ... is open to talk with me about difficult or taboo topics. | |  | | O | |  | | O | |  | | O | |  | | O | |  | | O | |
|  | ... has good social skills and offers me social support (e.g. involvement of relatives). | |  | | O | |  | | O | |  | | O | |  | | O | |  | | O | |
|  | ... offers me emotional support when I need it (in dealing with my feelings). | |  | | O | |  | | O | |  | | O | |  | | O | |  | | O | |

| **D3.** | **Are you currently seeing a neurologist?**  *Please select only one of the following answers.* |
| --- | --- |
|  |  |
| O | Yes |
| O | No |
|  | *Any complementary comments on your selection?* |

| 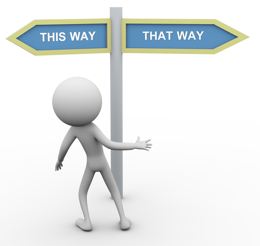 | *If* ***yes****🡪 continue with question* ***D4***  *If* ***no****🡪 continue with question* ***E1*** |
| --- | --- |

| **D4.** | **Are you getting the emotional support you want from your neurologist (e.g., through listening, encouragement, or empathy)?**  Please mark an X on the scale at the point where you rate your emotional support and then write the number in the comment field. 0 means no emotional support at all, 10 means the best possible emotional support. |
| --- | --- |

|  | \| **0** \|  \|  \|  \|  \|  \|  \|  \|  \| **10** \| \| --- \| --- \| --- \| --- \| --- \| --- \| --- \| --- \| --- \| --- \| \|  \|  \|  \|  \|  \|  \|  \|  \|   *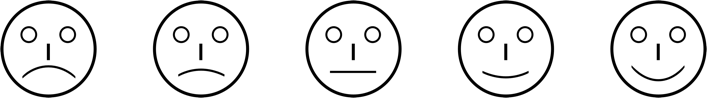*  *What number would the set cross correspond to?*  *Please specify here.*   \|  \|  \| \| --- \| --- \|   *Describe the emotional support you would like to receive from your treating neurologist. Please enter your answer in the lines below.* |
| --- | --- | --- | --- | --- | --- | --- | --- | --- | --- | --- | --- | --- | --- | --- | --- | --- | --- | --- | --- | --- | --- |

| **D5.** | **What aspects of your neurologist's support do you value most and why?**  *Please enter your answer in the lines below.* |
| --- | --- |

|  | _________________________________________________________________ |
| --- | --- |
|  | _________________________________________________________________ |
|  | _________________________________________________________________ |
|  | _________________________________________________________________ |
|  | _________________________________________________________________ |
|  | _________________________________________________________________ |
|  | _________________________________________________________________ |

| **D6.** | **Would you like to receive any other forms of support from your neurologist?**  *Please enter your answer in the lines below.* |
| --- | --- |

|  | _________________________________________________________________ |
| --- | --- |
|  | _________________________________________________________________ |
|  | _________________________________________________________________ |
|  | _________________________________________________________________ |
|  | _________________________________________________________________ |
|  | _________________________________________________________________ |
|  | _________________________________________________________________ |

**Part E: General information**

*Below are some questions about MS disease progression and therapies
over the* ***past six months.***

| **E1.** | **Please indicate the current progression type of your MS disease.**  *Please select only one of the following answers.* |
| --- | --- |
|  |  |
| O | Clinically isolated syndrome (CIS) / no definite MS diagnosis |
| O | Shear MS (RRMS) |
| O | Primary progressive MS (PPMS) |
| O | Secondary progressive MS (SPMS) |
| O | Transition between two stages or other forms (please specify below). |
|  | *Any complementary comments on your selection?* |
|  | |

| **E3.** | **How many different immunomodulatory drugs have you received in the last six months? If none, indicate 0.**  *Only numbers may be entered in this field.*   \| *Ex.* \| 2 \| Quantity \|  \| \| --- \| --- \| --- \| --- \| |
| --- | --- | --- | --- | --- | --- |
| \|  \|  \| Quantity \|  \| \| --- \| --- \| --- \| --- \| | |

|  |
| --- |

| 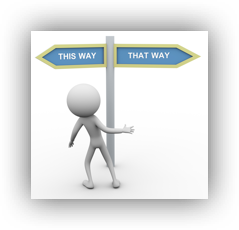 | *If* ***at least 1 drug*** *--> continue with question* ***E4*** *(next page).*  *If* ***no drug*** *--> skip question* ***E4*** *(end of questionnaire.* |
| --- | --- |

| **E4.** | **Which one(s) of the following immunomodulatory drugs have you received in the last six months? Please also indicate if you have stopped or interrupted any immunomodulatory medication in the last year.**  *Please select the applicable answers for the corresponding drugs (multiple answers possible).* |
| --- | --- |

|  | |  | **In the last six months**  **get** |  | **In the last six months**  **stopped or interrupted** |
| --- | --- | --- | --- | --- | --- |
|  | Betaferon® / interferon beta 1b |  | O |  | O |
|  | Extavia® / interferon beta 1b |  | O |  | O |
|  | Avonex® / interferon beta 1a |  | O |  | O |
|  | Rebif® / interferon beta 1a |  | O |  | O |
|  | Plegridy® / peginterferon beta 1a |  | O |  | O |
|  | Copaxone® / glatiramer acetate |  | O |  | O |
|  | Gilenya® / Fingolimod |  | O |  | O |
|  | Tysabri® / Natalizumab |  | O |  | O |
|  | Tecfidera® / BG-12 / Dimethyl fumarate |  | O |  | O |
|  | Aubagio® / teriflunomide |  | O |  | O |
|  | *Continuation of the drugs on the next page ...* |  |  |  |  |

|  | |  | **In the last six months**  **get** |  | **In the last six months**  **stopped or interrupted** |
| --- | --- | --- | --- | --- | --- |
|  | *Continuation of the medication...* |  |  |  |  |
|  | Nerventra® / Laquinimod |  | O |  | O |
|  | Lemtrada™ / alemtuzumab |  | O |  | O |
|  | Novantrone® / Mitoxantrone |  | O |  | O |
|  | Imurek® / Azathioprine |  | O |  | O |
|  | Synacthen® / Corticotropin |  | O |  | O |
|  | Sandimmun® / cyclosporine |  | O |  | O |
|  | Endoxan® / cyclophosphamide |  | O |  | O |
|  | MabThera® / rituximab |  | O |  | O |
|  | Ocrevus® / ocrelizumab |  | O |  | O |
|  | Mavenclad® / Cladribine |  | O |  | O |
|  | Vumerity™ / Diroximelfumarate |  | O |  | O |
|  | Other drug 1 |  | O |  | O |
|  | *------------------------------------------* |  |  |  |  |
|  | Other drug 2 |  | O |  | O |
|  | *------------------------------------------* |  |  |  |  |

You have now answered all the questions in this supplementary survey. Thank you very much.

By now, we have learned to live with the coronavirus for a while. Nevertheless, this pandemic can still burden us. We would therefore like to point out again where you can get help if necessary:

If you are looking for qualified and independent advice or helpful, supportive conversations, you can call the MS-Infoline of the Swiss. MS Society (0844 674 636). The specialists of the Swiss. MS Society are specialized in all questions and challenges concerning MS. The Swiss. MS Society is financed without any contribution from the pharmaceutical industry and also finances the MS Registry through donations.

Thank you very much for your commitment!

If you have any questions, please do not hesitate to contact us during office hours:

Tel.: 044 634 48 59

E-mail: ms-register@ebpi.uzh.ch

*Importance scales by subgroups*

**Figure 1** illustrates the distribution of importance ratings given by participants to services and infrastructure in practices, clinics, and rehabilitation centers by multiple sclerosis (MS) types. Participants receiving neurological care considered experience and expertise in MS care by healthcare professionals as the most important factors, with practical aspects like exchange of electronic health records being of lesser importance. However, for participants with progressive forms of MS, easy access to the practice or clinic was comparably more significant than for participants with relapsing-remitting MS (RRMS). **Figure 3** shows the distribution of importance participants attached to qualities and expertise of healthcare professionals (HCPs) involved in MS care. Both participants with RRMS, as well as for those with progressive MS ranked allocating adequate time, possessing specific expertise in MS care and communicating in a clear manner as the most important aspects of care provided by HCPs. On the other hand, the provision of emotional support was considered relatively less important in all three groups.

**Tables 1** and **2** display the mean scores of responses to each item on both Likert scales.


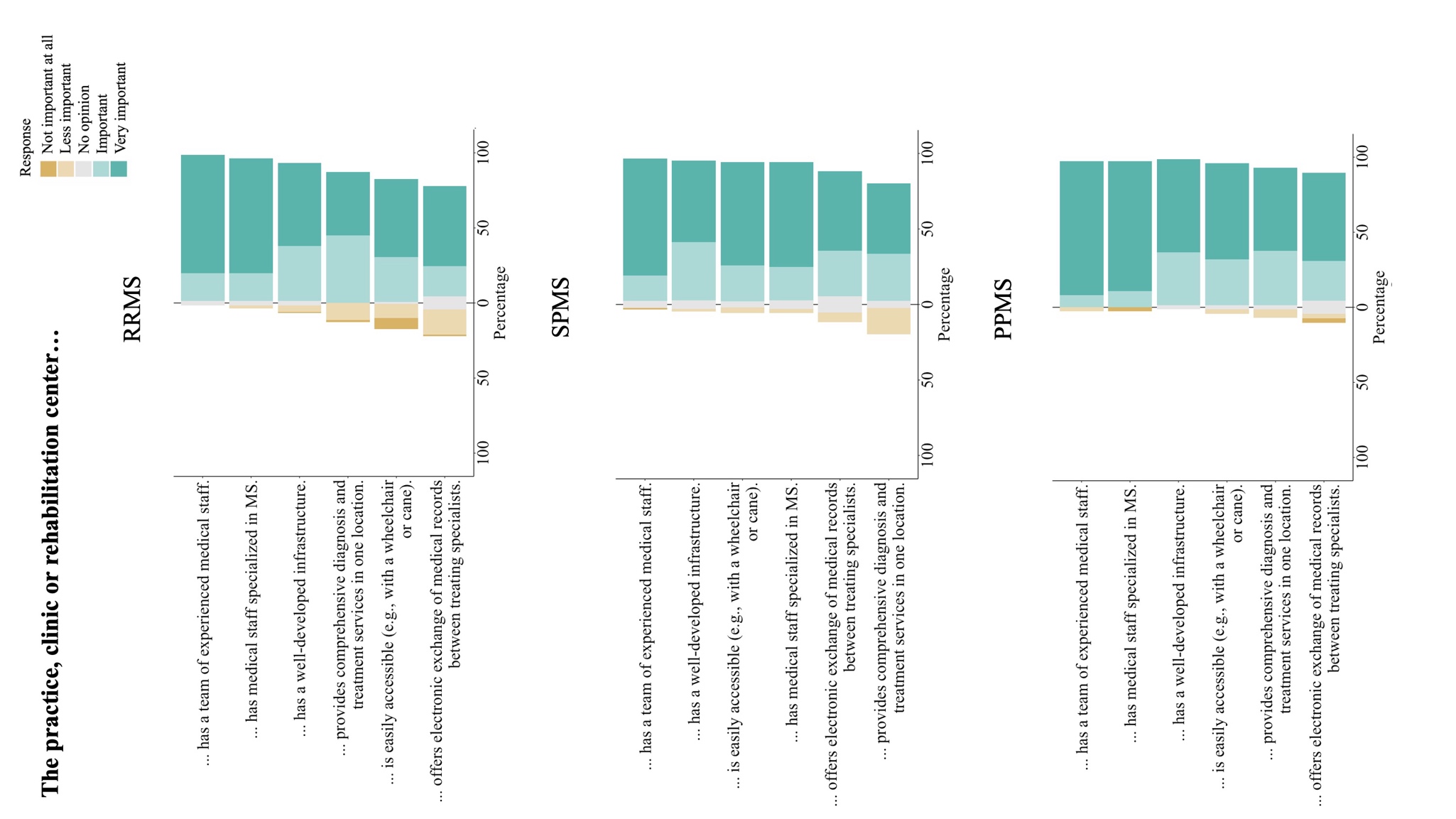


**Figure 1.** Participants’ ratings of the importance of services and infrastructure in practices, clinics and rehabilitation centers by MS types.


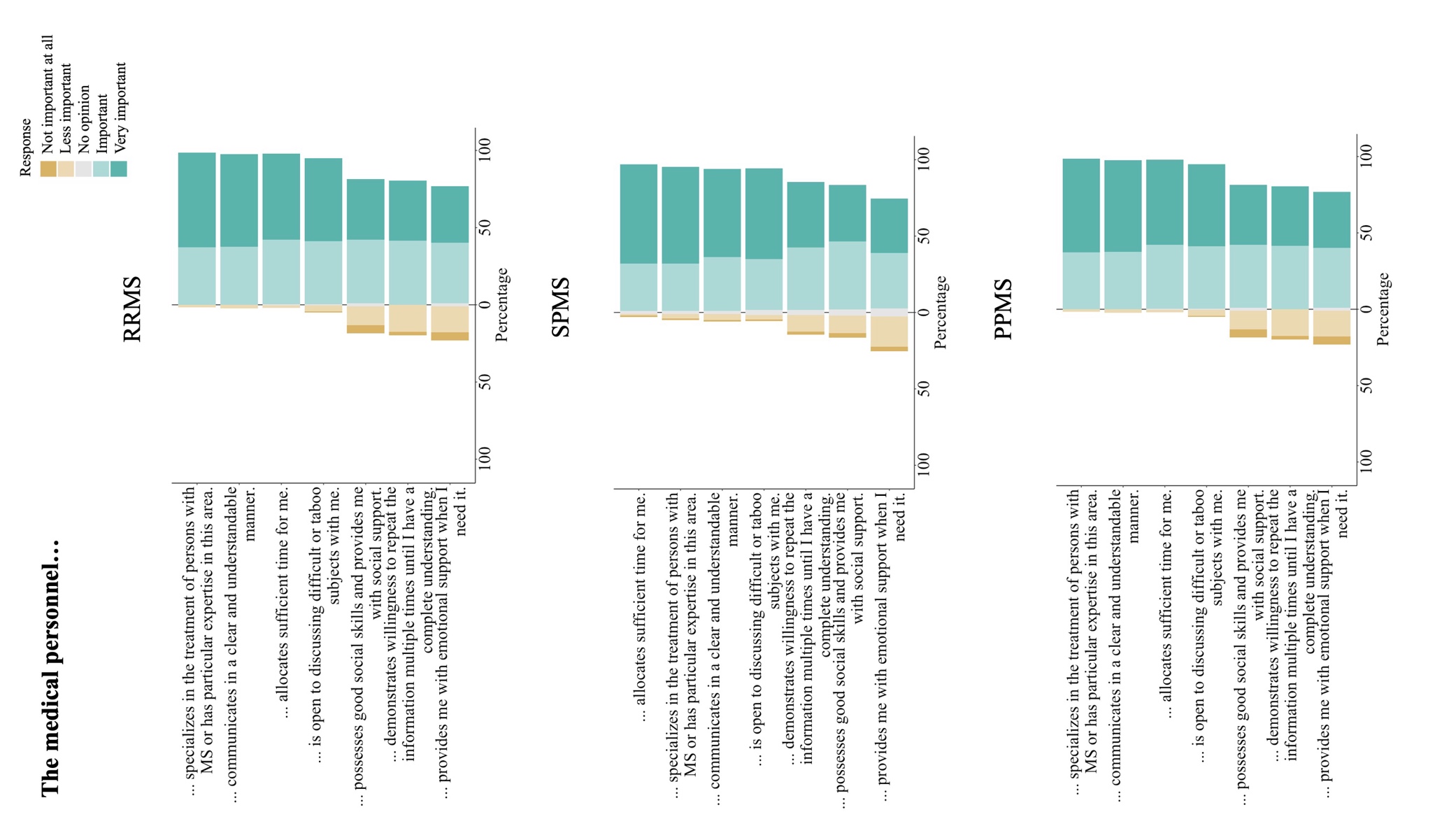


**Figure 2.** Participants’ ratings of the importance of the qualities and expertise of healthcare professionals in providing MS care by MS types.

**Table 1.** Mean scores for each item on the Likert scale assessing the importance of services and infrastructure in practices, clinics, and rehabilitation centers, categorized by subgroups of sex, age, MS duration, MS type, and gait disability.

| **Characteristic** | **Statement item** | | | | | |
| --- | --- | --- | --- | --- | --- | --- |
|  | **Access**  **Mean (SD)** | **Infrastructure**  **Mean (SD)** | **Centralized services**  **Mean (SD)** | **Use of EHR**  **Mean (SD)** | **Staff specialized in MS**  **Mean (SD)** | **Experienced staff**  **Mean (SD)** |
| **Sex** |  |  |  |  |  |  |
| Female | 4.41 (1.00) | 4.50 (0.68) | 4.17 (0.99) | 4.17 (1.09) | 4.65 (0.68) | 4.76 (0.56) |
| Male | 4.18 (1.13) | 4.35 (0.83) | 4.12 (1.07) | 4.24 (1.00) | 4.68 (0.69) | 4.72 (0.58) |
| **Age group** |  |  |  |  |  |  |
| 55 to 64 | 4.32 (1.05) | 4.46 (0.66) | 4.19 (1.00) | 4.18 (1.08) | 4.66 (0.68) | 4.76 (0.50) |
| 65 to 74 | 4.28 (1.04) | 4.3 (0.87) | 4.02 (1.03) | 4.23 (1.03) | 4.73 (0.58) | 4.72 (0.58) |
| 75 and more | 4.29 (1.07) | 4.36 (0.93) | 4.50 (0.65) | 4.50 (0.85) | 4.71 (0.61) | 4.93 (0.27) |
| **MS type** |  |  |  |  |  |  |
| CIS | 5.00 (0) | 5.00 (0) | 4.5 (0.71) | 3.50 (2.12) | 4.50 (0.71) | 5.00 (0) |
| PPMS | 4.50 (0.72) | 4.53 (0.57) | 4.38 (0.83) | 4.31 (1.00) | 4.75 (0.76) | 4.91 (0.30) |
| RRMS | 4.07 (1.25) | 4.4 (0.80) | 4.22 (0.91) | 4.12 (1.17) | 4.73 (0.55) | 4.75 (0.51) |
| SPMS | 4.53 (0.79) | 4.38 (0.72) | 4.00 (1.14) | 4.31 (0.89) | 4.59 (0.72) | 4.70 (0.57) |
| Transition | 4.00 (NA) | 4.00 (NA) | 4.00 (NA) | 4.00 (NA) | 5.00 (NA) | 5.00 (NA) |
| **MS duration group** |  |  |  |  |  |  |
| 1 to 9 | 4.19 (1.03) | 4.57 (0.51) | 4.19 (0.87) | 4.38 (0.97) | 4.48 (1.08) | 4.81 (0.40) |
| 10 to 19 | 4.36 (1.01) | 4.53 (0.55) | 4.28 (0.93) | 4.23 (1.05) | 4.78 (0.41) | 4.82 (0.42) |
| 20 to 29 | 4.15 (1.19) | 4.37 (0.84) | 4.22 (0.92) | 4.27 (0.95) | 4.68 (0.62) | 4.77 (0.53) |
| 30 to 39 | 4.50 (0.86) | 4.48 (0.63) | 4.10 (1.08) | 4.19 (1.11) | 4.74 (0.50) | 4.64 (0.62) |
| 40 and more | 4.47 (0.90) | 4.11 (0.99) | 4.00 (1.11) | 4.05 (1.18) | 4.42 (0.90) | 4.63 (0.68) |
| **Gait disability** |  |  |  |  |  |  |
| Mild | 4.02 (1.25) | 4.37 (0.76) | 4.09 (0.99) | 4.17 (1.11) | 4.64 (0.73) | 4.74 (0.54) |
| Moderate | 4.60 (0.66) | 4.49 (0.66) | 4.24 (0.95) | 4.24 (1.00) | 4.74 (0.49) | 4.77 (0.47) |
| Severe | 4.88 (0.34) | 4.44 (0.73) | 4.25 (1.06) | 4.44 (0.73) | 4.50 (0.89) | 4.94 (0.25) |

MS – multiple sclerosis, CIS – clinically isolated syndrome, PPMS – primary progressive multiple sclerosis, SPMS – secondary progressive multiple sclerosis, SD – standard deviation, EHR – electronic health record.

**Table 2.** Mean scores for each item on the Likert scale assessing the qualities and expertise of healthcare professionals in providing MS care, categorized by subgroups of sex, age, MS duration, MS type, and gait disability.

| **Characteristic** | **Statement item** | | | | | | |
| --- | --- | --- | --- | --- | --- | --- | --- |
|  | **Specialized in MS**  **Mean (SD)** | **Sufficient time**  **Mean (SD)** | **Speaks clearly**  **Mean (SD)** | **Repeats information**  **Mean (SD)** | **Taboo subjects**  **Mean (SD)** | **Social support**  **Mean (SD)** | **Emotional support**  **Mean (SD)** |
| **Sex** |  |  |  |  |  |  |  |
| Female | 4.53 (0.74) | 4.57 (0.62) | 4.48 (0.76) | 4.13 (1.04) | 4.40 (0.84) | 3.99 (1.14) | 3.98 (1.13) |
| Male | 4.59 (0.57) | 4.59 (0.57) | 4.49 (0.76) | 3.93 (1.15) | 4.46 (0.7) | 4.11 (1.04) | 3.59 (1.3) |
| **Age group** |  |  |  |  |  |  |  |
| 55 to 64 | 4.59 (0.68) | 4.58 (0.60) | 4.48 (0.81) | 4.10 (1.07) | 4.48 (0.79) | 4.08 (1.1) | 3.88 (1.19) |
| 65 to 74 | 4.45 (0.72) | 4.53 (0.65) | 4.47 (0.65) | 3.97 (1.12) | 4.30 (0.83) | 3.92 (1.15) | 3.72 (1.24) |
| 75 and more | 4.54 (0.52) | 4.77 (0.44) | 4.62 (0.51) | 4.08 (1.12) | 4.23 (0.6) | 3.85 (0.99) | 4.15 (1.14) |
| **MS type** |  |  |  |  |  |  |  |
| CIS | 3.50 (2.12) | 4.00 (0) | 4.50 (0.71) | 4.00 (0) | 4.00 (0) | 4.00 (0) | 4.00 (0) |
| PPMS | 4.66 (0.48) | 4.78 (0.42) | 4.41 (0.91) | 4.09 (1.12) | 4.22 (0.97) | 4.12 (1.04) | 3.84 (1.08) |
| RRMS | 4.61 (0.59) | 4.54 (0.55) | 4.56 (0.65) | 4.01 (1.12) | 4.46 (0.73) | 3.99 (1.19) | 3.86 (1.25) |
| SPMS | 4.47 (0.79) | 4.56 (0.70) | 4.43 (0.83) | 4.13 (1.04) | 4.45 (0.80) | 4.04 (1.05) | 3.85 (1.20) |
| Transition | 5.00 (NA) | 5.00 (NA) | 4.00 (NA) | 4.00 (NA) | 5.00 (NA) | 3.00 (NA) | 4.00 (NA) |
| **MS duration group** |  |  |  |  |  |  |  |
| 1 to 9 | 4.45 (0.94) | 4.45 (0.76) | 4.40 (0.75) | 4.15 (1.04) | 4.55 (0.51) | 4.25 (0.91) | 3.95 (0.94) |
| 10 to 19 | 4.70 (0.55) | 4.71 (0.46) | 4.60 (0.71) | 4.14 (1.08) | 4.43 (0.83) | 4.11 (1.12) | 3.99 (1.26) |
| 20 to 29 | 4.54 (0.68) | 4.58 (0.50) | 4.44 (0.82) | 4.03 (1.10) | 4.46 (0.82) | 4.03 (1.05) | 3.71 (1.16) |
| 30 to 39 | 4.50 (0.66) | 4.52 (0.66) | 4.50 (0.66) | 4.05 (1.03) | 4.39 (0.84) | 3.86 (1.21) | 3.7 (1.25) |
| 40 and more | 4.33 (0.91) | 4.48 (0.93) | 4.52 (0.93) | 4.1 (1.26) | 4.33 (0.91) | 3.9 (1.30) | 4.05 (1.36) |
| **Gait disability** |  |  |  |  |  |  |  |
| Mild | 4.55 (0.79) | 4.50 (0.69) | 4.50 (0.83) | 4.01 (1.11) | 4.45 (0.79) | 3.99 (1.19) | 3.82 (1.28) |
| Moderate | 4.56 (0.52) | 4.67 (0.50) | 4.42 (0.73) | 4.10 (1.08) | 4.33 (0.81) | 3.96 (1.06) | 3.87 (1.12) |
| Severe | 4.31 (0.79) | 4.56 (0.51) | 4.50 (0.52) | 4.25 (0.86) | 4.56 (0.63) | 4.31 (0.70) | 3.75 (1.06) |

MS – multiple sclerosis, CIS – clinically isolated syndrome, PPMS – primary progressive multiple sclerosis, SPMS – secondary progressive multiple sclerosis, SD – standard deviation.

*Frequency of observed support types*

A total of 296 codes, categorized as perceived support provided by a neurologist and distributed over five support dimensions, were found at least once per participant. At least one code pertaining to informational support appeared in the data of 123 participants, accounting for 41.6% of all unique codes per support type, thus making informational support the most frequently described category. There were 78 (26.4%) codes pertaining to emotional support, while the least commonly observed support dimension was tangible support, found the data of 17 participants, which corresponded to 5.7% of all unique codes. Both male and female participants most frequently mentioned receiving informational support from their neurologist, with emotional support as the second most common for both groups. When examining support types within MS type subgroups, informational support emerged as the most commonly mentioned type across all MS types, at approximately 40%. Notably, participants with PPMS mentioned tangible support more frequently compared to participants with other forms of MS support, with percentages of 13.8% compared to 4.3% and 5.8% among participants with RRMS and SPMS, respectively (**Table 3**).

**Table 3.** Frequency of codes of various types of perceived support by sex and MS type, with each support type mention counted only once per participant.

| **Type of support** | **Sex** | | **MS Type** | | | **Total**  **N = 296** |
| --- | --- | --- | --- | --- | --- | --- |
|  | **Female**  **N = 204** | **Male**  **N = 92** | **PPMS**  **N = 29** | **RRMS**  **N = 162** | **SPMS**  **N = 104** |  |
| **Informational** | 77 (37.7%) | 46 (50.0%) | 11 (37.9%) | 66 (40.7%) | 45 (43.3%) | 123 (41.6%) |
| **Tangible** | 9 (4.4%) | 8 (8.7%) | 4 (13.8%) | 7 (4.3%) | 6 (5.8%) | 17 (5.7%) |
| **Esteem** | 20 (9.8%) | 1 (1.1%) | 1 (3.4%) | 18 (11.1%) | 2 (1.9%) | 21 (7.1%) |
| **Network** | 42 (20.6%) | 15 (16.3%) | 5 (17.2%) | 31 (19.1%) | 21 (20.2%) | 57 (19.3%) |
| **Emotional** | 56 (27.5%) | 22 (23.9%) | 8 (27.6%) | 40 (24.7%) | 30 (28.8%) | 78 (26.4%) |

MS – multiple sclerosis, RRMS – relapsing-remitting multiple sclerosis, PPMS – primary progressive multiple sclerosis, SPMS – secondary progressive multiple sclerosis

A total of 73 mentions were categorized as unmet support needs. With codes present in the data of 36 (49.3%) participants, the most commonly observed unmet need overall was for informational support. This was followed by emotional and network support, each mentioned 11 times (15.1%). Mentions by men were predominantly related to informational support (N = 18, 78.3%), whereas there was a more balanced distribution of mentions across all support types among women. One-fifth of all mentions by women pertained to emotional support, while the percentage was lower among men (4.3%). When codes were grouped by MS type, participants with SPMS expressed more unmet needs than those with RRMS. Examining subgroups of codes created by participants with different MS types, unmet needs for informational support were the most commonly observed across all MS type subgroups (**Table 4**).

**Table 4**. Frequency of codes of unmet needs for different support types by sex and MS type, with each support type mention counted only once per participant.

| **Type of support** | **Sex** | | **MS Type** | | | **Total**  **N = 73** |
| --- | --- | --- | --- | --- | --- | --- |
|  | **Female**  **(N = 50)** | **Male**  **(N = 23)** | **PPMS**  **(N = 9)** | **RRMS**  **(N = 27)** | **SPMS**  **(N = 37)** |  |
| **Informational** | 18 (36.0%) | 18 (78.3%) | 5 (55.6%) | 12 (44.4%) | 19 (51.4%) | 36 (49.3%) |
| **Tangible** | 5 (10.0%) | 4 (17.4%) | 2 (22.2%) | 3 (11.1%) | 4 (10.8%) | 9 (12.3%) |
| **Esteem** | 6 (12.0%) | 0 | 0 | 4 (14.8%) | 2 (5.4%) | 6 (8.2%) |
| **Network** | 11 (22.0%) | 0 | 2 (22.2%) | 3 (11.1%) | 6 (16.2%) | 11 (15.1%) |
| **Emotional** | 10 (20.0%) | 1 (4.3%) | 0 | 5 (18.5%) | 6 (16.2%) | 11 (15.1%) |

MS – multiple sclerosis, RRMS – relapsing remitting-multiple sclerosis, PPMS – primary progressive multiple sclerosis, SPMS – secondary progressive multiple sclerosis

Finally, out of 21 participants who explicitly stated that they did not need emotional support, the majority of participants had RRMS (N = 12, 57.1%), six participants (28.6%) had SPMS and three (14.3%) had PPMS. Out of these participants, 11 (52.4%) were women and 10 (47.6%) were men, which constitutes 7.9% of women and 14.1% of all men who provided at least one open-ended response.
